# Acupoint-Specific, Frequency-Dependent, and Improved Insulin Sensitivity Hypoglycemic Effect of Electroacupuncture Applied to Drug-Combined Therapy Studied by a Randomized Control Clinical Trial

**DOI:** 10.1155/2014/371475

**Published:** 2014-06-16

**Authors:** Rong-Tsung Lin, Chung-Yuh Tzeng, Yu-Chen Lee, Ying-I Chen, Tai-Hao Hsu, Jaung-Geng Lin, Shih-Liang Chang

**Affiliations:** ^1^Department of Internal Medicine and Emergency Medicine, Division of Endocrinology and Metabolism, Tungs' Taichung Metro Harbor Hospital, Taichung County 43503, Taiwan; ^2^Department of Occupational Safety and Health, Jen-Teh Junior College of Medicine, Nursing and Management, Houlong, Miaoli County 35664, Taiwan; ^3^Department of Orthopedics, Taichung Veterans General Hospital, Taichung 40705, Taiwan; ^4^Institute of Molecular Medicine, College of Life Science, National Tsing Hua University, Hsinchu 30013, Taiwan; ^5^Department of Acupuncture, China Medical University Hospital, Taichung 40402, Taiwan; ^6^Department of Medicinal Botanicals and Health Application, Da-Yeh University, No. 168, University Road, Dacun, Changhua County 51591, Taiwan; ^7^School of Chinese Medicine, China Medical University, Taichung 40402, Taiwan

## Abstract

The application of electroacupuncture (EA) to specific acupoints can induce a hypoglycemic effect in streptozotocin-induced rats, normal rats, and rats with steroid-induced insulin resistance. EA combined with the oral insulin sensitizer rosiglitazone improved insulin sensitivity in rats and humans with type II diabetes mellitus (DM). There are different hypoglycemic mechanisms between Zhongwan and Zusanli acupoints by EA stimulation. On low-frequency (2 Hz) stimulation at bilateral Zusanli acupoints, serotonin was involved in the hypoglycemic effect in normal rats. Moreover, after 15 Hz EA stimulation at the bilateral Zusanli acupoints, although enhanced insulin activity mainly acts on the insulin-sensitive target organs, the muscles must be considered. In addition, 15 Hz EA stimulation at the bilateral Zusanli acupoints has the combined effect of enhancing cholinergic nerve activity and increasing nitric oxide synthase (NOS) activity to enhance insulin activity. Despite the well-documented effect of pain control by EA in many systemic diseases, there are few high-quality long-term clinical trials on the hypoglycemic effect of EA in DM. Combination treatment with EA and other medications seems to be an alternative treatment to achieve better therapeutic goals that merit future investigation.

## 1. Introduction

Acupuncture is a complementary and alternative therapy that is based on the yin and yang theory [1]. The therapeutic goal of acupuncture is to regulate “*Qi* and blood” to achieve better health condition. Acupuncture regulates the* Qi-*blood balance and likely adjusts the blood flow throughout the body, influences absorption, or regulates the disturbance of* Qi* under disease conditions [2]. According to the Traditional Chinese Medicine (TCM) theory, different acupoints have different therapeutic effects [1]. Under the guide of meridian theory, physician can use different acupoints to treat disease following the meridian pathway distant from the focus of disease. Numerous clinical utilizations of acupuncture and planned studies have been developed since the mid-twentieth century. The greatest positive research outcomes were achieved in the areas of pain management [3], stroke-induced paralysis management [4], antiemesis treatment [5], and drug addiction treatment [6].

Electroacupuncture (EA), which applies an adequate electrical current via needles to acupoints to produce responses, was recently developed. The aim of EA is to apply continuous electrical current stimulation to the needle throughout treatment. Except the same points are stimulated during treatment, this technique is particularly helpful in anesthesia and in the treatment of pain and stroke. Unlike traditional acupuncture, this device can adjust the frequency and intensity of impulse, depending on the severity of diseases. Besides, one advantage of EA is that different frequency of EA had different therapeutic effect on pain relief [7]. In addition to the effect on pain relief after lower abdominal surgery by EA, there are also frequency-dependent effects on postoperative analgesic agents requirements with low-frequency (2 Hz) compared to high-frequency EA (100 Hz). In the field of EA on insulin sensitivity, there are areas such as improvement in glucose tolerance in which EA was able to achieve improved results of insulin sensitivity in basic research and clinical trials [8–14]. Also, some studies showed that different frequencies of EA stimulation (10 and 100 Hz) can change the energy metabolism and lower down plasma glucose levels in induced hyperglycemic rats [15]. So far, there are no review articles to summarize if EA had the frequency-dependent hypoglycemic effect as the effect on pain relief. This systemic review is conducted to illustrate that EA had the acupoint-specific and/or frequency-dependent hypoglycemic activity and improved insulin sensitivity, especially applying in combined with oral hypoglycemic agents, such as rosiglitazone for controlling the hyperglycemic state in patient with diabetes [14, 16].

The use of combination therapy over monotherapy is increasing in the treatment of many systemic diseases such as rheumatoid arthritis, diabetes mellitus (DM), and hypertension because of their complex pathological factors. Drug interactions in combination therapy were studied previously. The combination therapy of efficacy and safety has also been shown in Western clinical studies of the treatment of many systemic diseases. As with osteoarthritis, pharmacological management is often ineffective, and agents such as analgesics may cause harmful side effects. Acupuncture seems to provide pain relief as an adjunctive therapy for osteoarthritis of the knee compared with control group treatment [24]. In other studies, acupuncture plus medications showed better effect on total sleep duration than medications alone [25]. In stroke rehabilitation, the study findings indicated that combining EA with strength training treatment reduced muscle spasticity and may have improved the motor function of chronic stroke survivors with moderate or severe muscle spasticity [4].

DM is a complicated metabolic disorder that presents as abnormally high blood glucose levels or impaired glucose tolerance. Hyperinsulinemia and insulin resistance are two important pathogeneses of type II DM that may further progress to pancreatic failure [26]. For treatment, the usual species of combination drug treatment of type II DM were sulfonylureas and the class of thiazolidinediones (TZD), which may directly decrease insulin resistance by enhancing insulin action within the skeletal muscle, liver, and adipose tissue [27]. Although the efficacy of long-term combination drug treatment of type II DM has improved, the drug treatment effect worsens in these patients over time. Since the ability of EA to improve glucose tolerance and insulin activity was shown in basic research and clinical trials [8, 9, 14], the combination of the benefit of the insulin-enhancing activity of EA with other oral hypoglycemic agents can thus be considered an alternative method for treating DM [16].

Although EA has been widely used in many clinical conditions as mentioned above, the benefit of its application as routine treatment for DM or as adjunctive therapy to improve glucose tolerance is unknown. Meanwhile, the findings of various basic studies and clinical trials regarding the interaction between EA and oral hypoglycemic agents when used as combination therapy need to be further discussed and analyzed. Besides, it is important to determine whether EA enhances or weakens the effect of concurrently used oral hypoglycemic agents. That is, are there opportunities to use combination therapy to improve insulin activity and regulate the secretion of insulin to minimize the overall impact on pancreatic *β*-cell function?

## 2. Materials and Methods

Here, we conducted a systematic review of the treatment effects of EA on hypoglycemic activity and impaired glucose tolerance as well as on insulin activity in different types of rodent models of diabetes and in patients with DM and its interaction with concurrently used oral hypoglycemic agents.

This review also analyzed both basic research and clinical trials on the ability of EA to improve impaired glucose tolerance and determine its effects on the pathophysiology of different types of rodent models and humans with diabetes in studies published in English in the PubMed database from 1999 to 2013. The inclusion criteria of references were also considered that were focused on the frequency-dependent, acupoint-specific, and/or drug-combined effect on the regulation of plasma glucose levels in EA therapy.

## 3. Results

### 3.1. Hypoglycemic Effect of EA on the Zhongwan/Gwanyuan Acupoint

The pathogenesis of type II DM is complex and involves the interaction of genetic and environmental factors that are characterized mainly by insulin resistance and pancreatic *β*-cell failure. In humans, the complex interaction between multiple susceptible genes and the environment makes the genetic analysis and pathogenesis research of diabetes difficult. Rodent models of DM have great advantages over human studies in discerning the pathogenesis in DM because of their relatively short generation length and small size.

Acupuncture at the Zhongwan/Gwanyuan acupoint has been widely used in TCM to relieve symptoms of DM. A decrease in plasma glucose levels was observed in rats after EA (15 Hz, 10 mA) for 30 min at the Zhongwan/Gwanyuan acupoint [17]. This was observed in normal rats and rat models with type II (neonatal streptozotocin- (STZ-) induced noninsulin dependent) DM. No significant effect on plasma glucose levels was observed in rat models with genetically derived BioBreeding type I (insulin-dependent) diabetes. Insulin-like immunoreactivity in the plasma of normal and type II diabetic rats was greatly increased by EA stimulation at the Zhongwan/Gwanyuan acupoint compared with the basal concentrations. That is, EA stimulation at the Zhongwan/Gwanyuan acupoint can induce a marked reduction in plasma glucose levels in rat models with preserved pancreatic function; thus, an insulin-dependent action can be considered. However, EA stimulation at the Zhongwan/Gwanyuan acupoint cannot reduce plasma glucose levels in type II diabetic rats with higher insulin resistance induced by repeated injection of insulin. Thus, we hypothesized that higher insulin resistance may exist in this type of rodent model by daily injection of long-acting human insulin that results in poor response to insulin-like immunoreactivity substances endogenously secreted by EA stimulation at the Zhongwan/Gwanyuan acupoint or exogenous insulin.

It has also been mentioned that EA applied at different frequencies can stimulate the release of *β*-endorphin to activate specific opioid receptors in the pancreas [28] that stimulate insulin secretion [29]. Therefore, we suggest that EA stimulation at the Zhongwan/Gwanyuan acupoint induces secretion of endogenous *β*-endorphin, which reduces plasma glucose concentrations in an insulin-dependent manner. *β*-endorphin secretion can activate opioid *μ*-receptors, thereby resulting in increased glucose transporter isoform protein (GLUT4) expression and induced insulin secretion via activation of opioid receptors in pancreatic *β*-cells, and may modulate its secretion [30, 31]. To trace the source of *β*-endorphin, one study showed that EA at 2 Hz for 30 min in rats decreased plasma glucose levels, an effect that could be abolished by naloxone. A similar effect of EA was also observed in wild-type mice but disappeared in *μ*-opioid receptor knockout mice. Mediation of the *μ*1-opioid receptor is thought to result from blockade of the response to EA by naloxonazine in rats. Otherwise, adrenalectomy abolished not only the hypoglycemic response to EA in rats and mice but also the increase in plasma *β*-endorphin and insulin levels by EA in rats [19]. In conclusion, the increase in plasma *β*-endorphin levels by EA simulation at the Zhongwan/Gwanyuan acupoint at 2 Hz frequency is mainly derived from the adrenal gland.

According to the meridian theory of TCM, different acupoints have different effects. To compare the effects of different acupoints on hypoglycemia in experimental ADX and control rats, 2 Hz EA was applied to the Zhongwan/Gwanyuan or bilateral Zusanli acupoints [19]. In the experimental group, the hypoglycemic effect disappeared after stimulating the Zhongwan/Gwanyuan acupoint with 2 Hz EA and partial lowering plasma glucose was observed at the bilateral Zusanli acupoint with the same frequency of stimulation; furthermore, plasma *β*-endorphin and insulin levels showed no significant changes after stimulation of both acupoints. In normal Wistar rats, naloxone can completely block the hypoglycemic effect of the Zhongwan/Gwanyuan acupoint but only partially block the effect of the Zusanli acupoint stimulated by 2 Hz EA. Thus, we speculate that the hypoglycemic effect of 2 Hz EA at the Zhongwan/Gwanyuan is derived from the pathway of endogenous opioid peptide effect via insulin secretion [19]. Besides, the partial hypoglycemic effect of EA stimulation at the bilateral Zusanli acupoints with the same frequency may have another mechanism excluding the endogenous opioid peptide pathway that will be discussed later.

One study showed that stimulation by low-frequency (2 Hz) EA at the Zhongwan/Gwanyuan acupoint less effectively produced a hypoglycemic effect than that at a higher frequency (15 Hz) [19]. In addition, different frequencies of EA have been reported to activate different subtypes of opioid receptors [2, 32]. By studying the role of the adrenal gland in the hypoglycemic response to high-frequency (15 Hz) EA stimulation at the Zhongwan/Gwanyuan acupoint in ADX normal rats, we found a sharper decrease in the plasma glucose levels by higher frequency EA stimulation in the fasting sham-operated group than in the fasting ADX group [20]. Naloxone blocked this hypoglycemic response to EA stimulation in ADX rats. Stimulation of EA failed to elicit an increase in plasma *β*-endorphin or insulin levels in ADX rats. Similar results were observed in sham and ADX mice. Furthermore, naloxone abolished the hypoglycemic response to EA stimulation in mice. Such a hypoglycemic response to EA stimulation was also observed in *μ*-opioid receptor knockout mice (MOR-KOM). Thus, mediation by another opioid peptide from other organs or the brain should be considered as in the earlier study, which reported an increase in EOP levels in the brain of ADX rats [33].

Studies have also shown that different frequencies of EA can induce different kinds of neuropeptide secretion from the central nervous system (CNS). In addition, a better treatment effect was found by the combination use of different frequencies of EA that resulted in the synchronous release of all opioid peptides [20, 34]. Other clinical trials also showed that EA had a frequency- (dose) dependent pain relief effect after lower abdominal surgery. The antiemetic effect and total opioid amount requirement were decreased as the dosage of EA increased from 2 Hz to 100 Hz [7]. In summary, a frequency-dependent hypoglycemic activity effect of EA stimulation at the Zhongwan/Gwanyuan acupoint should be considered, since higher frequency (15 Hz) EA stimulation can induce secretion of endogenous opioid peptides from multiple sites to decrease the plasma glucose level [20] (Table 1).

### 3.2. Hypoglycemic Effect of EA on the Zusanli Acupoints

The Zusanli acupoint has been traditionally shown to alter intestinal motility within the digestive system according to the 12 regular channels of the meridian theory that may influence the carbohydrate metabolic rate [35]. According to Chang and colleagues, the hypoglycemic effect of EA was much greater in rats stimulated at the Zusanli acupoint (ST36) than in rats stimulated at the Zhongwan acupoint [19]. However, no significant hypoglycemic response was found after 2 Hz EA stimulation at the Zusanli acupoint in STZ-induced insulin-dependent DM (IDDM) rats after the use of opioid receptor blockers (naloxone) and serotonin depleters (*p*-chlorophenylalanine; PCPA) to explore the mechanism of the hypoglycemic effect of EA at both the Zusanli acupoints in normal rats. The hypoglycemic effect of this EA was not completely blocked by naloxone. That is, other substances or transmitters may mediate the hypoglycemic response to 2 Hz EA stimulation at the bilateral Zusanli acupoints by mechanisms other than the EOP pathway. In addition, PCPA treatment did not reproduce a hypoglycemic response to 2 Hz EA in naloxone-treated rats or MOR-KOM. However, the direct injection of serotonin significantly decreased the plasma glucose levels [21]. Thus, serotonin must play an important role in the hypoglycemic action of 2 Hz EA stimulation at both the Zusanli acupoints in normal rats (Table 1).

### 3.3. Effect of EA on Insulin Sensitivity

Insulin resistance, defined as an impaired biological response to either exogenously or endogenously derived insulin, can impair insulin action on insulin-sensitive target organs and cause impaired glucose tolerance as seen in DM. Through an intravenous glucose tolerance test (ivGTT) and insulin challenge test (ICT), 15 Hz EA stimulation at the Zusanli acupoints was used in normal Wistar and STZ-induced IDDM rats to evaluate its effect on insulin sensitivity. In addition to the effect of this stimulation on the hypoglycemic activity in normal Wistar rats, it induced hypoglycemic activity in insulin-deficient STZ-induced IDDM rats after stimulation at the bilateral Zusanli acupoints. EA improved the glucose tolerance during an ivGTT, and significant improvement in the Homeostasis model assessment (HOMA) index [36] was found in the experimental group compared with the control group [8]. Interestingly, endogenous plasma insulin levels showed no difference between the experimental and control groups in normal Wistar rats.

During an ICT, exogenous insulin activity was enhanced as more hypoglycemic activity was found in normal Wistar and insulin-deficient STZ diabetic rats in the experimental group than in the control group. Thus, 15 Hz EA stimulation at the bilateral Zusanli acupoints can improve glucose tolerance by enhancing insulin sensitivity in rats. Further studies were planned to explore the mechanism by which EA stimulation at the Zusanli acupoint improves insulin resistance from the viewpoint of molecular biology and to examine the neuroendocrine effect and its relationship to insulin signal proteins. Higher insulin resistance was induced by the use of large steroid (prednisolone) doses in normal rats, which manifested as elevated HOMA index, higher levels of free fatty acids (FFA), and impaired glucose tolerance. The use of 15 Hz EA stimulation at the bilateral Zusanli acupoints can improve glucose tolerance and insulin sensitivity by decreasing serum FFA levels [11]. The insulin-signaling proteins IRS1 and GLUT4 that were inhibited by the steroids in skeletal muscle recovered after this EA.

Both sympathetic and parasympathetic nerves are innervated in muscles, adipose tissue, the liver, and the pancreas. Imbalance of neuroendocrine activity can alter carbohydrate metabolism. For example, hyperactive sympathetic tone can induce an insulin antagonist effect that results in insulin resistance. In addition, sympathetic activity can inhibit the antilipolytic effect of insulin, which can increase FFA release, whereas parasympathetic activity can decrease FFA production by adipose tissue. Elevated sympathetic activity relative to parasympathetic activity can induce a higher FFA release from adipose tissue and result in decreased insulin-stimulated glucose uptake in target organs that contribute to the development of insulin resistance. Studies have also shown that EA can inhibit sympathetic activity by regulating NOS expression in the CNS [37] and stimulating the parasympathetic nerves, which decreases hepatic glucose release [12], using cholinergic inhibitors and NOS antagonists to explore its mechanism in normal rats by EA stimulation at the Zusanli acupoint to improve glucose tolerance. The data showed that insulin activity was enhanced by EA in normal male Wistar rats via increased neuronal NOS (nNOS) activity and upregulation of the insulin-signaling protein IRS1 expression in the rat skeletal muscle [22]. Thus, EA has the combined effect of enhancing cholinergic nerve activity and increasing nNOS activity via decreasing plasma FFA concentrations to improve glucose tolerance by facilitating insulin activity.

Another recent study also showed that the hypoglycemic effect of EA stimulation at the Zusanli (ST-36) acupoint resulted from stimulation of the cholinergic nerve in STZ-induced insulin-dependent rat models of DM. In that study, the Zusanli (ST-36) acupoint also stimulated the expression of insulin signaling proteins IRS1 and AKT2, and atropine treatment blocked the EA-induced expression of those insulin-signaling proteins [23]. Studies have also shown that bilateral cervical vagotomy of the liver can induce insulin resistance and that this phenomenon could be reversed by acetylcholine, which stimulates hepatic insulin-sensitizing substance (HISS) secretion to improve peripheral tissue insulin activity [38]. Thus, we assumed that activated cholinergic nerve activity and increased acetylcholine secretion may play part in the hypoglycemic response to 15 Hz EA stimulation at the bilateral Zusanli acupoints. The dual hypoglycemic effects on both parasympathetic nerve and neurotransmitters (acetylcholine, NOS) during EA stimulation at the Zusanli acupoints should be considered.

**Figure 1:**
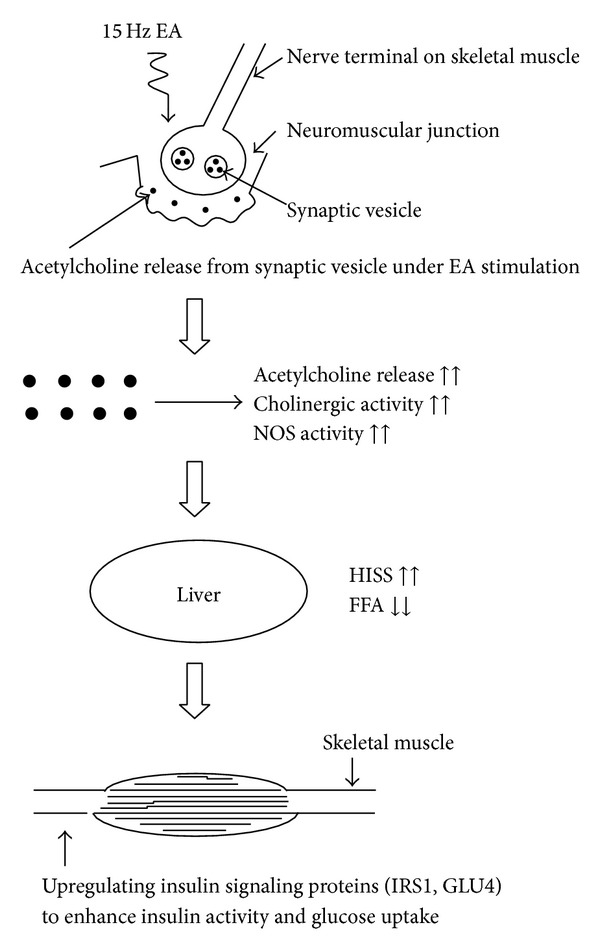
Schematic proposed mechanism of 15 Hz electroacupuncture (EA) stimulation at the Zusanli acupoints to improve glucose tolerance and insulin activity through acetylcholine release, activate cholinergic nerves, and increase the effect of nitric oxide synthase (NOS) on skeletal muscles.

Acetylcholine and NOS may serve as first messengers to affect target cells in the liver via blood flow to influence secondary HISS release. That is, large amounts of acetylcholine may be released from the nerve ending vesicles on the neuromuscular junctions during EA manipulation under continuous electric current stimulation. These neurotransmitters have the same effect as cholinergic nerves that enhance NOS activity to decrease plasma FFA concentrations. Simultaneously, via the bloodstream into the portal venous flow, acetylcholine stimulated HISS release from the liver. HISS may then act on the insulin-sensitive target organs to improve insulin resistance in peripheral tissues, including muscles, by upregulating insulin-signaling proteins (IRS1, GLUT4) to increase glucose uptake (Figure 1). As mentioned before, the hypoglycemic effect of EA may consist of regulating or modifying* Qi* and blood to achieve the treatment effect. From the physiological point of view,* Qi* and blood may be linked with the nervous system and neurotransmitters. Its meaning in ancient China can be explained scientifically on the bases of physiology to clarify this phenomenon.

### 3.4. Clinical Trials and Combination Effect of EA with Other Medications

Although EA had a hypoglycemic effect in experimental diabetes models by increasing insulin secretion via release of *β*-endorphins from multiple sources or enhancing insulin activity, little evidence and a lack of randomized control trials observing the clinical applications of EA to improve insulin activity in humans have been seen. The most well-known and documented analgesic effect of acupuncture is relief of multiple pain syndromes such as osteoarthritis and migraine by *β*-endorphin or endomorphin secretion. The concept of using EA as a new modality for combination treatment with other medications is based on the observations of the effects of many combination treatments on chronic diseases (e.g., combining sulfonylureas and metformin to treat DM or angiotensin-receptor blockers with diuretics to treat hypertension) to achieve better therapeutic outcomes.

To date, no studies published in the PubMed database have explored the effect of EA on plasma glucose levels of patients with DM. The approach of using EA to treat other insulin-resistant conditions such as obesity seems to be a good reference for understanding the effect of EA on glucose metabolism and insulin activity. Earlier research showed that EA was an effective treatment for obesity and can decrease serum glucose levels by increasing insulin secretion [10].

According to the combination therapy concept [39], a randomized control trial (RCT) of patients with type II DM treated with rosiglitazone alone compared with rosiglitazone + EA therapy was designed to study its effect on plasma glucose level and influence on insulin activity. In this study, combined therapy can decrease hyperinsulinemia stress, which improves insulin activity evaluated by the HOMA index via reducing plasma FFA levels [14]. Although no significant hypoglycemic effect was seen after single-dose EA combination therapy compared with single rosiglitazone treatment alone, a long-term and large sample-containing RCT is needed to clarify the hypoglycemic effect of EA combined with different medications in patients with DM.

Transcutaneous electrical nerve stimulation (TENS) is a noninvasive alternative treatment for pain relief similar to EA in which electrical currents pass through the skin on conducting pads [40]. An earlier study showed that the application of TENS to the ST36 and SP6 acupoints can prevent hyperglycemic responses during anesthesia. Researchers found that TENS can significantly decrease plasma glucose levels, insulin levels, and the HOMA index in the experimental group compared with the placebo group after the use of TENS during preoperative elective hysterectomy [13]. Thus, TENS may be considered a new treatment modality for plasma glucose control and insulin activity enhancement before surgery.

## 4. Discussion and Summary

**Figure 2:**
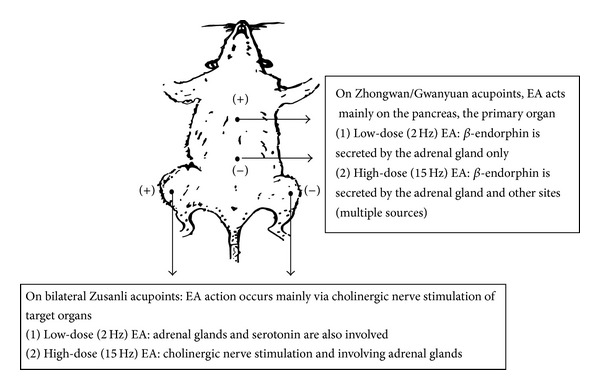
Schematic presentation of the acupoint-specific frequency-dependent hypoglycemic effect of electroacupuncture (EA) stimulation at specific acupoints. (a) EA stimulation at the Zhongwan/Gwanyuan acupoint, the insulin-dependent hypoglycemic effect which mainly affects the pancreas, the primary endogenous insulin-secreting organ. (b) 15 Hz EA stimulation at the bilateral Zusanli acupoints improved insulin sensitivity mainly by affecting the muscles, insulin-sensitive target organs.

In summary, here we hypothesized an acupoint-specific and/or dose (frequency)-dependent hypoglycemic effect of EA at specific acupoints (Figure 2). In EA stimulation at the Zhongwan acupoint, an insulin-dependent hypoglycemic effect mainly from the action of endogenous insulin secreted from the primary organ, the pancreas, should be considered. In low-dose stimulation (2 Hz) at the Zhongwan acupoint, increased secretion of endogenous *β*-endorphin only from the adrenal gland activates opioid *μ*-receptors on pancreatic *β*-cells, which contribute to the hypoglycemic effect by increasing insulin secretion. In higher dose stimulation (15 Hz) at the Zhongwan acupoint, endogenous opioid peptides secreted from sites other than the adrenal gland help lower the plasma glucose levels by increasing insulin secretion. In addition, after 15 Hz EA stimulation at the bilateral Zusanli acupoints, improved insulin sensitivity and/or increasing insulin-hypoglycemic activity should be considered to predominantly affect insulin-sensitive target organs such as the muscles. That is, 15 Hz EA stimulation at the bilateral Zusanli acupoints has the combined effect of enhancing cholinergic nerve activity and increasing NOS activity via lowering of plasma FFA concentrations to improve glucose tolerance through the upregulation of insulin-signaling proteins to facilitate insulin activity.

In the past 10 years, a few clinical studies on EA and its effect on insulin-resistance-related disease have been published in English in the PubMed database. To date, all the randomized control clinical trials had small patient numbers and relatively short treatment durations that may have led to inappropriate interpretation of their results. EA combination therapy seems to relieve stress-related hyperinsulinemia by improving insulin resistance and reducing plasma FFA levels. An RCT with a large sample size should be conducted and more efforts must be made to clarify the hypoglycemic effect or enhance insulin activity on EA combination treatment with different medications in patients with DM. The above evidence suggests that EA may be a component for combination therapy or have the potential advantage of prolonging the timing of pancreatic *β*-cell failure on specific acupoints by decreasing insulin secretion via enhancing insulin sensitivity in patients with DM.

## Figures and Tables

**Table 1:** Basic study of the hypoglycemic effect of EA and its influence on insulin activity.

Model categories on different acupoints	Animal models	EA dosage/duration	Conclusion	Reference
EA stimulation at the Zhongwan/Gwanyuan acupoint	Normal rat Neonatal STZ-induced type II diabetic rats Genetically derived type I IDDM rats STZ induced adult type I diabetic rats	15 Hz, 10 mA for 30 min	Electroacupuncture stimulation at the Zhongwan acupoint induces secretion of endogenous *β*-endorphin, which reduces plasma glucose concentrations in an insulin-dependent manner.	[17]
	*μ*-Opioid receptor knockout mice Wild-type mice ADX rats	2 Hz, 10 mA for 30 min	Activation of the *μ*-opioid receptor by *β*-endorphin secreted from the adrenal gland to release insulin resulting in the decrease of plasma glucose. Mediation of the mu(1) opioid receptor is considered.	[18]
	ADX rats Normal rats	2 Hz, 10 mA for 30 min	Hypoglycemic effect came from adrenal gland, followed by endogenous opioid peptide (EOP) pathway, affected by insulin secretion after 2 Hz EA stimulation at the Zhongwan/Gwanyuan acupoint. Only partial hypoglycemic effect on 2 Hz EA bilateral Zusanli acupoints came from the adrenal gland.	[19]
	*μ*-Opioid receptor knockout mice Wild-type mice ADX rats	15 Hz, 10 mA for 30 min	Multiple sources of endogenous opioid peptide participated in the lowering of plasma glucose in rats induced by EA stimulation at higher frequency (15 Hz) at the Zhongwan/Gwanyuan acupoint.	[20]

EA stimulation at the Zusanli acupoint	STZ-induced IDDM rats Wild-type mice *μ*-Opioid receptor knockout mice Normal rats	2 Hz, 10 mA for 30 min	Serotonin also involved in the hypoglycemic action of 2 Hz EA stimulation at both the Zusanli acupoints of normal rats	[21]
	STZ-induced IDDM rats	15 Hz, 10 mA for 30 min	15 Hz EA on bilateral Zusanli acupoints in rats improved glucose tolerance and lowered plasma glucose levels. Hypoglycemic effects of exogenous insulin were enhanced in normal Wistar rats and STZ diabetic rats.	[8]
	Steroid-induced insulin resistant rats	15 Hz, 10 mA for 30 min	Insulin resistance was successfully induced by a large dose of prednisolone in male rats. This insulin resistance can be improved by 15 Hz EA on the bilateral Zusanli acupoints via decreased plasma levels of FFAs.	[11]
	Normal rats	15 Hz, 10 mA for 60 min	EA stimulated cholinergic nerves and nitric oxide synthase to lower plasma FFA levels and improve glucose tolerance.	[22]
	STZ-induced insulin dependent non-ADX and ADX diabetic rats	15 Hz, 10 mA for 30 min	15 Hz EA stimulation at the Zusanli acupoints induced a hypoglycemic response in STZ-induced diabetic rats by stimulating the cholinergic nerves and involving the adrenal glands, which in turn stimulates the expression of insulin-signaling proteins.	[9, 11, 23]

EA: electroacupuncture; STZ: streptozotocin; IDDM: insulin-dependent diabetes mellitus; ADX: adrenalectomized; FFAs: free fatty acids.

## Abbreviations

STZ: Streptozotocin
ADX rats: Adrenalectomized rats
IDDM: Insulin-dependent diabetes mellitus
EA: Electroacupuncture
ivGTT: Intravenous glucose tolerance test
ICT: Insulin challenge test
EOP: Endogenous opioid peptide
FFA: Free fatty acid
GLUT4: Glucose transporting protein 4
HISS: Hepatic insulin-sensitizing substance
TCM: Traditional Chinese medicine
DM: Diabetes mellitus.
